# Patient-Derived Breast Cancer Bone Metastasis *In Vitro* Model Using Bone-Mimetic Nanoclay Scaffolds

**DOI:** 10.1155/2023/5753666

**Published:** 2023-03-11

**Authors:** Haneesh Jasuja, Farid Solaymani Mohammadi, Jiha Kim, Anu Gaba, Dinesh R. Katti, Kalpana S. Katti

**Affiliations:** ^1^Department of Civil Construction and Environmental Engineering, North Dakota State University, Fargo, ND 58108, USA; ^2^Department of Biological Sciences, North Dakota State University, Fargo, ND 58108, USA; ^3^Sanford Roger Maris Cancer Center, Fargo, ND 58102, USA

## Abstract

The unavailability of reliable models for studying breast cancer bone metastasis is the major challenge associated with poor prognosis in advanced-stage breast cancer patients. Breast cancer cells tend to preferentially disseminate to bone and colonize within the remodeling bone to cause bone metastasis. To improve the outcome of patients with breast cancer bone metastasis, we have previously developed a 3D *in vitro* breast cancer bone metastasis model using human mesenchymal stem cells (hMSCs) and primary breast cancer cell lines (MCF-7 and MDAMB231), recapitulating late-stage of breast cancer metastasis to bone. In the present study, we have tested our model using hMSCs and patient-derived breast cancer cell lines (NT013 and NT023) exhibiting different characteristics. We investigated the effect of breast cancer metastasis on bone growth using this 3D *in vitro* model and compared our results with previous studies. The results showed that NT013 and NT023 cells exhibiting hormone-positive and triple-negative characteristics underwent mesenchymal to epithelial transition (MET) and formed tumors in the presence of bone microenvironment, in line with our previous results with MCF-7 and MDAMB231 cell lines. In addition, the results showed upregulation of Wnt-related genes in hMSCs, cultured in the presence of excessive ET-1 cytokine released by NT013 cells, while downregulation of Wnt-related genes in the presence of excessive DKK-1, released by NT023 cells, leading to stimulation and abrogation of the osteogenic pathway, respectively, ultimately mimicking different types of bone lesions in breast cancer patients.

## 1. Introduction

Breast cancer is a leading cause of cancer-related deaths in women worldwide [1], causing fatal skeletal failure at their advanced stage [2]. Invasive breast cancer is the most common cancer affecting women in the United States, and 287,850 individuals are expected to be diagnosed in 2022. Of these, 43,250 are estimated to die from it in 2022 [3]. Breast cancer metastasis accounts for the majority of deaths from breast cancer. Bone is a common site of metastases for breast cancer [4] and can cause significant complications such as pain, pathologic fracture, hypercalcemia, and spinal cord compression [5, 6]. These complications can also lead to death from breast cancer. Detection of breast cancer and treatment of metastasis at the earliest stage is important to decrease mortality [7]. Metastatic breast cancer is still an incurable disease. Median survival is about 3–5 years for hormonal receptor-positive metastatic breast cancer [8, 9] and 1–2 years for triple-negative metastatic breast cancer [10, 11]. Due to complex cellular heterogeneity within cancer cells [12] and the low success rate of novel drugs for metastasized breast cancer in clinical trials [13], effective treatment for advanced-stage breast cancer remains a challenge for researchers. New models are required wherein a personalized approach to selecting the best treatment for a patient can be determined in a timely manner. Using patient-derived breast cancer cell lines to create 3D *in vitro* models for personalized drug selection in the treatment of metastatic breast cancer is a major step forward in this direction.

While some preclinical models, such as two-dimensional (2D) monolayer cell culture models and *in vivo* mice models, have been utilized by researchers for preclinical cancer research, these have several limitations. 2D models are known to poorly recapitulate the *in vivo* complexity due to a lack of cell-microenvironment interactions, while *in vivo* models often fail to develop into metastatic disease [14]. Thus, there is a need to create new robust preclinical models that better recapitulate human tumor biology at their advanced stage and can be used for high-throughput drug screening. Increasing evidence has shown that three-dimensional (3D) disease models derived from patients' own healthy and tumor tissue could better predict the pathogenesis of cancer cells and provide a more accurate measurement of potential drugs than existing models because these models retain characteristic features of cancer cells derived from individual patient's cells [15]. Despite classifying breast cancer cells into three categories based on their cell surface receptors and growth behavior, breast cancer patients within each category can have markedly different disease outcomes and therapeutic responses [16]. Thus, models derived from patients' cancer cells could help researchers better predict therapeutic responses.

Several attempts at developing 3D *in vitro* models of metastasized breast cancer have been made [17–21]. However, such models have utilized only cancer cells to resemble the metastatic stage. In addition, efforts have been made to recapitulate bone metastasis of breast cancer by coculturing breast cancer cells with osteoblasts [22–24]. However, such models failed to mimic the ideal *in vivo* conditions of breast cancer metastasis to bone due to inaccurate representation of the bone microenvironment where cancer cells interact with remodeled bone. Thus, to address this issue, we developed a novel 3D *in vitro* bone metastatic scaffold model using a tissue-engineered approach, where bone marrow-derived hMSCs differentiate into bone cells and generate extracellular matrix (ECM) for breast cancer dissemination to better recapitulate breast cancer bone metastasis [25, 26]. These bone metastatic scaffolds possess high porosity (86.1%) with a pore size range between 100 and 300 *μ*m and exhibit a high compressive modulus of 2.495 MPa, essential for hard tissue growth [27]. Previously, we utilized primary human breast cancer cells-MCF-7 and MDAMB231 to develop this 3D *in vitro* breast cancer bone metastasis model [26] and investigated the role of the Wnt/*β*-catenin pathway in osteogenic differentiation of hMSCs on the scaffold surface during breast cancer bone metastasis [28].

The present study aims to understand the metastases of patient-derived breast cancer cells to bone and their role in hMSCs osteogenic differentiation. We evaluated the effect of cancer on bone growth via the Wnt/*β*-catenin pathway and compared our results with previous studies.

## 2. Materials and Methods

### 2.1. Preparation of Polycaprolactone (PCL)-*in situ* Hydroxyapatite (HAP) Clay Scaffolds

PCL-*in situ* HAPclay scaffolds were prepared per the procedure described previously [27, 29–32]. Sodium-montmorillonite (Na-MMT) clay was received from the Clay Minerals Society (Wyoming). Briefly, PCL-*in situ* HAPclay scaffolds were prepared using the freeze-drying method by mixing 10% *in situ* HAPclay with PCL (Sigma Aldrich) in 1,4-dioxane (Sigma Aldrich). HAP was biomineralized into intercalated nanosheets of MMT clay due to increased d-spacing between sheets by 5-aminovaleric acid (Sigma–Aldrich) modifiers, resulting in the modification of Na-MMT clay to *in situ* HAPclay. Finally, 12 mm diameter and 3 mm thick cylindrical scaffolds were used for the experiments.

### 2.2. Cell Lines and Cell Culture

**Figure 1:**
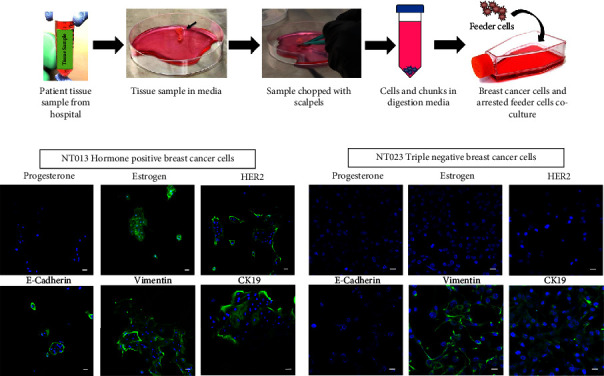
(a) Schematic showing the steps in isolation of breast cancer cells from the patient tissue samples (b), (c) representative immunofluorescence microscope images of NT013 and NT023 cells cultured in 2D culture. Scale bar: 20 *μ*m. *n* = 3 (b) and (c).

Human mesenchymal stem cells (hMSCs) were purchased from Lonza (PT-2501) and cultured in MSCGM BulletKit medium (Lonza, PT3001). Human breast cancer cell lines NT013 and NT023 were derived from the patient tissue samples obtained from Sanford Roger Maris Cancer Center, Fargo. The ethical committee approved the study, and before surgery, all patients provided written informed consent to allow any excess tissue to be used for research. If there was extra breast cancer tissue that would not be needed for clinical diagnosis and management, it was submitted for the study to the NDSU team for further study. The tissue samples were excised from the primary site (breasts) of patients. After surgery, the pathologist reviewed the excised breast tissue to confirm the presence of cancer cells. The cancer cells present in NT013 and NT023 breast tissue specimens were characterized as hormone-positive (ER/PR positive) and triple-negative (ER/PR/HER2 negative), respectively, by the clinical pathologist. Patient tissue samples were transported to the research lab using a transportation medium containing DMEM, 1% of pen/strep mix (100x), gentamicin (10 mg/ml), and amphotericin B (250 *μ*g/ml). Breast cancer cells were isolated using a cell isolation kit (Miltenyi Biotec- 130-095-929) following the manufacturer's protocol and cocultured with irradiated 3T3-J2 feeder cells (Kerafast) (Figure 1(a)). Finally, cells were maintained at 37°C and 5% CO_2_ in high glucose DMEM containing 5 *μ*g/ml insulin, 250 ng/ml amphotericin B, 10 *μ*g/ml gentamicin, 0.1 nM cholera toxin, 0.125 ng/ml epidermal growth factor (EGF), 25 ng/ml hydrocortisone, ROCK inhibitor Y-27632 10 *μ*M, 10% (v/v) FBS, and 100 U/mL penicillin and 100 *μ*g/mL streptomycin.

### 2.3. Cell Seeding

**Figure 2:**
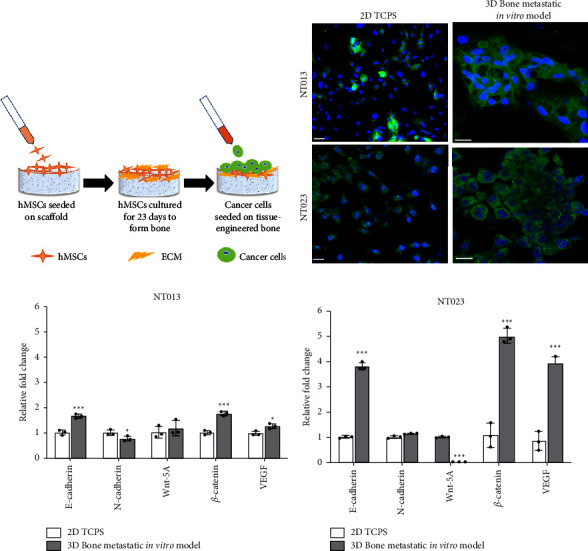
(a) Schematic showing steps of sequential culture. (b) Tumor morphology determined by staining cancer cells with EpCAM. Scale bar: 20 *μ*m. (c) Quantified gene expressions of E-cadherin, N-cadherin, Wnt-5A, *β*-catenin, and VEGF in NT013 cells under different conditions. ^*∗*^*p* < 0.05 and ^*∗∗∗*^*p* < 0.001 indicate a significant difference between NT013 cells grown on 2D TCPS and NT013 cells grown on 3D Bone metastatic *in vitro* model. (d) Quantified gene expressions of E-cadherin, N-cadherin, Wnt-5A, *β*-catenin, and VEGF in NT023 cells under different conditions. ^*∗∗∗*^*p* < 0.001 indicate a significant difference between NT023 cells grown on 2D TCPS and NT023 cells grown on 3D Bone metastatic *in vitro* model. The fold change for gene expression is relative to the 2D condition. *n* = 3 (c) and (d).

Scaffolds were sterilized in 70% ethanol for 24 hours, further sterilized under UV light for 45 min, washed twice in phosphate-buffered saline (PBS), and finally immersed in the culture medium and incubated for 24 hours in a humidified 5% CO_2_ incubator at 37°C. hMSCs were seeded at a density of 1 × 10^5^ cells per scaffold and cultured for 23 days to obtain tissue-engineered bone on the scaffold surface. Next, patient-derived breast cancer cells were seeded at a density of 1 × 10^5^ cells per scaffold on the tissue-engineered bone and maintained in the breast cancer cells medium (Figure 2(a)). The media was changed every two days during both hMSCs and sequential culture of breast cancer cells on the scaffold surface.

### 2.4. Immunofluorescence Staining

Both 8-well chambers (Thermo scientific) seeded and scaffold seeded cells were washed twice in PBS and fixed in 4% paraformaldehyde (PFA) for 30 min. Next, cells were permeabilized with 0.2% TritonX-100 in PBS for 5 min, followed by blocking with blocking buffer (0.2% fish skin gelatin (FSG) with 0.02% Tween20) for 1 hour. Furthermore, the cells were incubated with the primary antibody overnight at 4°C. The primary antibodies were diluted in the blocking buffer using dilutions given in Table S1. Finally, cells were incubated with conjugated secondary antibodies corresponding to the species of used primary antibodies at 1 : 200 dilutions and incubated for 45 minutes at room temperature (RT). The nuclei were counterstained with 4,6-diamidino-2-phenylindole (DAPI), and immunofluorescence images were taken under a confocal microscope (Zeiss Axio Observer Z1 LSM 700).

### 2.5. Gene Expression by RT-qPCR

RNA was isolated from cells grown on TCPS (2D) and scaffolds using a Direct-zol RNA MiniPrep kit (Zymo Research) following the protocol described elsewhere [33]. Briefly, 1000 ng of RNA was reversed transcribed to cDNA using random primers and M-MLV reverse transcriptase (Promega) in a thermal cycler using a thermal profile- 70°C for 5 minutes (Applied Biosystems). Next, the qPCR experiment was performed using a 7500 Fast Real-Time PCR instrument (Applied Biosystems) using a thermal profile with a holding stage (5 min at 95°C) and a cycling stage (40 cycles of 30 s at 95°C and 1 min at 55°C). The mRNA expressions of genes (listed in Table S2) were quantified using their respective primers and normalized to the housekeeping gene glyceraldehyde-3-phosphate dehydrogenase (GAPDH). Finally, fold change in target gene expressions was calculated using the comparative Ct method (2^−ΔΔCt^).

### 2.6. ELISA Assays

**Figure 3:**
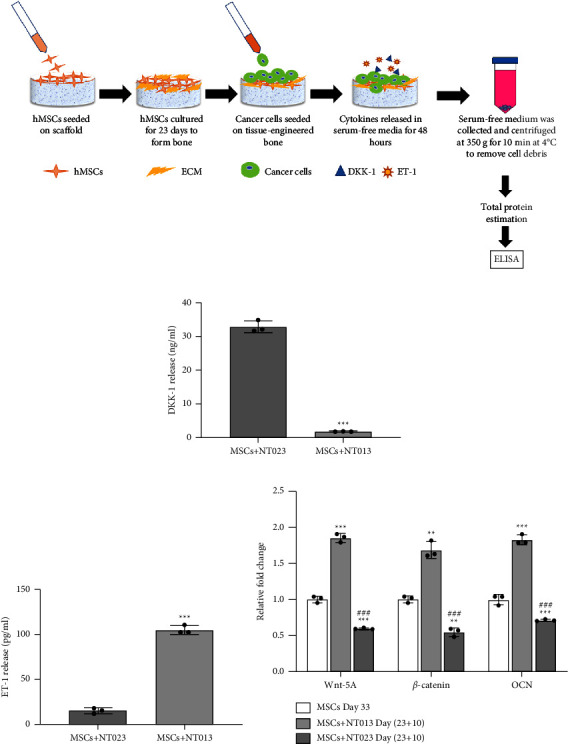
Breast cancer-released cytokines DKK-1 and ET-1 regulate the Wnt/*β*-catenin pathway: (a) schematic showing steps for harvesting cytokines from the sequential cultures; (b) quantified serum levels of DKK-1; (c) ET-1 by ELISA. ^*∗∗∗*^*p* < 0.001 indicate a significant difference between cytokines levels in MSCs + NT023 conditioned medium and MSC + NT013 conditioned media; (d) quantified gene expressions of Wnt-5a, *β*-catenin, and OCN. ^*∗∗*^*p* < 0.01 and ^*∗∗∗*^*p* < 0.001 indicate a significant difference between MSCs cultured with MSC + NT013 conditioned media for day (23 + 10) and control MSCs day 33, and MSCs cultured with MSC + NT023 conditioned media for day (23 + 10) and control MSCs day 33. ^###^*p* < 0.001 indicate significant difference between MSCs cultured with MSC + NT013 conditioned media for day (23 + 10) and MSCs cultured with MSC + NT023 conditioned media for day (23 + 10). The fold change for gene expression is relative to the control MSCs day 33. *n* = 3(b), (c), and (d).

The released DKK-1 and ET-1 cytokines concentration was measured in serum-free cell culture media using high-sensitivity ELISA kits of DKK-1 (RayBiotech) and ET-1 (RayBiotech) as per the manufacturer's protocol. The cell-seeded scaffolds were kept in a serum-free medium for 48 hours before collecting the medium for sample preparation (Figure 3(a)). Next, the medium was centrifuged at 350 × g for 10 minutes at 4°C to remove cell debris, and supernatants were stored at −20°C until analysis.

### 2.7. Statistical Analysis

Data were presented as the mean value ± standard deviation. Statistical significance between the two groups was determined by an unpaired Student's *t*-test or one-way or two-way ANOVA followed by Tukey's post-test using GraphPad Prism v7.04 software. The significance level was set at *p* ≤ 0.05. “*n*” represents the technical replicates of each experiment.

## 3. Results

### 3.1. Isolated Patient-Derived Cancer Cells Retained Their Idiosyncratic Characteristics

We assessed the characteristic proteins of breast cancer cells by immunostaining to confirm that cancer cells isolated from patient tissue samples retain their tumor-associated features. Breast cancer cells are broadly classified into three categories; hormone-positive, triple-negative, and HER2-positive, based on the expression of hormone receptors-estrogen (ER) and progesterone (PR) and human epidermal growth factor receptor 2 (HER2) [16]. After characterizing NT013 patient-derived cells, we observed that NT013 retained their hormone-positive characteristics by expressing ER. Interestingly, we also observed positive HER2 expression in NT013 cells. However, other studies on NT013 patient tissue by pathologists confirmed reduced HER2 levels (data not shown), suggesting that NT013 cells can be categorized into hormone-positive breast cancer cells. Next, we evaluated similar protein expression in NT023 cells and observed that NT023 cells do not express ER, PR, and HER2 receptors, retaining their triple-negative characteristics.

Cytokeratin-19 (CK19) is also a suitable marker for identifying breast cancer cells [34, 35]. CK19 is an epithelial cell marker, and its expression is seen in more than 90% of breast cancer cases. It is also reported that luminal-type hormonal positive cells exhibit higher positive rates of CK19 than triple-negative cells [34, 36]. We observed protein expression of CK19 in both cell lines; however, the expression was more intense in NT013 cells compared to NT023 cells. Next, we analyzed Epithelial to Mesenchymal transition (EMT) markers for NT013 and NT023 cells to identify their invasive nature. Epithelial cells can be identified by expressing the epithelial protein E-cadherin on their cell surface. In contrast, mesenchymal cells can be identified by various protein expressions such as N-cadherin, vimentin, and twist [37]. We observed that NT013 cells expressed both E-cadherin and vimentin protein expressions, while NT023 cells mostly expressed high vimentin levels, indicating that NT023 cells are more mesenchymal in nature compared to NT013 cells (Figure 1).

### 3.2. Bone Microenvironment-Induced MET in Breast Cancer Cells

EMT/MET processes represent the invasiveness of cancer cells where cancer cells leave their primary site to acquire migratory phenotype during EMT. At the same time, MET potentiates cancer cells to regain their epithelial characteristics and adapt to the new environment at their secondary site [38–40]. To investigate the effect of the bone microenvironment on patient-derived breast cancer cells' invasiveness, we analyzed their mRNA levels related to EMT/MET biomarkers such as E-cadherin and N-cadherin and compared our results with cells grown on a 2D surface. E-cadherin is a cell surface protein that participates in forming homotypic junctions across epithelial cells [41]. The loss of E-cadherin, while the gain in N-cadherin levels is associated with the EMT process of cancer cells [37], and vice-versa is valid for the MET process [39]. Previously, we observed that the primary breast cells- MCF-7 and MDAMB231 underwent mesenchymal to epithelial transition due to upregulation of E-cadherin and downregulation of vimentin and twist levels in the presence of bone [26]. Likewise, we observed a significant increase in E-cadherin levels (^*∗∗∗*^*p* < 0.001) in NT013 (∼2-fold) and NT023 (∼4-fold) breast cancer cells grown in the bone microenvironment compared to their respective 2D cell cultures. We also observed significant downregulation (^*∗*^*p* < 0.05) in N-cadherin levels in NT013 cells and insignificant change in N-cadherin levels in NT023 cells, indicating that both NT013 and NT023 cells acquired more epithelial characteristics in the bone microenvironment (Figure 2).

### 3.3. The Bone Microenvironment Induces Aggressiveness and Angiogenesis in Patient-Derived Cells

Wnt5a is an important member of the Wnt pathway and acts as either tumor-suppressive or tumor-promoting in different cancer types [42]. Lower levels of Wnt-5A expression are significantly associated with poor prognosis and more aggressive behavior in triple-negative breast cancer [43, 44]. Similarly, *β*-catenin is highly expressed in breast cancer patients [45] and is significantly associated with poor clinical outcomes in invasive breast cancer [46]. To evaluate the aggressive behavior of breast cancer cells in the presence of bone, we quantified their Wnt-5A and *β*-catenin levels. Our results showed significant downregulation (^*∗∗∗*^*p* < 0.001) in Wnt-5A levels in NT023 growing on the bone compared to 2D culture, while we didn't observe any significant change in Wnt-5A in NT013 cells. However, we observed significant upregulation (^*∗∗∗*^*p* < 0.001) in *β*-catenin levels in both NT023 and NT013 cells, indicating increased aggressiveness in the presence of bone.

VEGF is a well-known marker of angiogenesis, highly expressed in many solid tumors resulting in a poor prognosis of the disease [47]. We observed upregulation in the VEGF mRNA levels in both NT013 (^*∗*^*p* < 0.05) and NT023 (^*∗∗∗*^*p* < 0.001) cells grown on bone compared to their 2D cultures, respectively, indicating increased angiogenesis in both cell types in the presence of bone (Figure 2).

### 3.4. Tumor Formation by Patient-Derived Cell Lines on Bone Niche

To investigate the morphology of cancer cells on tissue-engineered bone after MET, we stained the cells with cancer-specific protein, EpCAM, and compared our results with cells grown on a 2D surface. EpCAM is a transmembrane protein significantly overexpressed in breast cancer tissues [48]. We observed that both NT013 and NT023 cell lines formed tumors on tissue-engineered bone. In contrast, cancer cells in their monoculture did not form any tumors. We also noticed that NT013 cells formed compact tumors on the bone microenvironment, exhibiting distinguishable cellular boundaries with strong cell-cell interactions, while NT023 cancer cells grouped into clusters (with moderate cell-cell interactions) instead of forming compact tumors. Previously, we observed that hormone-positive MCF-7 cells formed dense tumors on bone scaffolds [26], similar to NT013 breast cancer cells. In contrast, triple-negative MDAMB 231 cells formed loose aggregates (with poor cell-cell interaction) [26], different from NT023 cells (Figure 2(b)), indicating that inherent characteristic differences among two different triple-negative cells could alter their tumor-forming ability after interacting with the bone microenvironment.

### 3.5. DKK-1 and ET-1 Factors Released by Breast Cancer Cells Regulate the Osteogenic Wnt/*β*-Catenin Pathway

ET-1 and DKK-1 are well-known markers of bone metastases that induce osteoblastic or osteolytic lesions, respectively, in breast cancer patients resulting in the poor mechanical stability of the bone [49, 50]. It is reported that serum DKK-1 levels are higher in patients with bone metastasized breast cancer than at other metastatic sites [51]. Previously, we observed that MCF-7 cells grown on bone released high levels of ET-1 in serum-free media, whereas MDAMB231 cells released high levels of DKK-1, leading to stimulation and inhibition of osteogenesis via the Wnt/*β*-catenin pathway [25]. To investigate the effect of NT013 and NT023 released cytokines on bone health via Wnt signaling, we first quantified ET-1 and DKK-1 levels in the sequential culture of NT013 and NT023 and observed that NT013 cells released high levels of ET-1 (^*∗∗∗*^*p* < 0.001) while NT023 cells released high levels of DKK-1 (^*∗∗∗*^*p* < 0.001) in line with our results with MCF-7 and MDAMB231 cell lines, respectively. Next, to determine that ET-1 and DKK-1 released by patient-derived cells are involved in the regulation of the Wnt/*β*-catenin signaling mediated bone osteogenesis, we analyzed the expressions of Wnt-related genes of MSCs on Day (23 + 10) maintained under different conditioned mediums. We observed upregulation in both Wnt 5a (^*∗∗∗*^*p* < 0.001) and *β*-catenin (^*∗∗*^*p* < 0.01) expressions in hMSCs cultured with conditioned media of sequentially cultured NT013 cells containing high ET-1 levels. In contrast, hMSCs cultured with conditioned media of sequentially cultured NT023 cells containing high DKK-1 levels showed downregulation of Wnt 5a (^*∗∗∗*^*p* < 0.001) and *β*-catenin (^*∗∗*^*p* < 0.01) levels compared to control MSCs samples (Day 33). We also assessed the expression of a late-stage osteogenic marker, osteocalcin (OCN) in hMSCs cultured under different conditioned media w.r.t to control samples. We noticed a significant increase (^*∗∗∗*^*p* < 0.001) in mRNA levels of OCN in hMSCs cultured with conditioned media of sequentially cultured NT013 cells while downregulation in OCN levels (^*∗∗∗*^*p* < 0.001) in hMSCs cultured with conditioned media of sequentially cultured NT023 cells. Overall, the results suggested that cytokines released by NT013 cells stimulate Wnt/*β*-catenin signaling in hMSCs while cytokines released by NT023 cells abrogate the Wnt/*β*-catenin pathway, thus promoting and inhibiting osteogenesis, respectively (Figure 3).

## 4. Discussion

Many 3D *in vitro* models have been developed to recapitulate breast cancer bone metastasis disease conditions [52]. However, existing breast cancer bone metastatic models attempted to mimic late-stage breast cancer by coculturing breast cancer cells with osteoblasts that do not resemble the ideal conditions of breast cancer metastasis to bone *in vivo* [22–24]. In these coculture systems, different cell types were seeded together on the scaffold surface; however, in ideal conditions, breast cancer cells migrate to the remodeling bone, where cancer cells interact with differentiated bone cells and bone microenvironment to disseminate. Our 3D *in vitro* breast cancer bone metastatic model accurately represents the late stage of breast cancer metastasis to the bone, where we implemented a sequential culture system (Figure 2(a)). In the sequential culture, hMSCs differentiated into osteoblastic lineage on a nano clay-based scaffold along with calcium deposition [53] and collagen formation [27], thus generating a remodeling bone microenvironment for breast cancer metastasis. Previously, we have successfully developed a 3D *in vitro* bone metastatic model using primary breast cancer cell lines—MCF-7 and MDAMB231 [26]. Our results showed that MCF-7 and MDAMB231 breast cancer cells underwent MET and formed tumors in the bone microenvironment. Moreover, their interaction with bone cells induces the release of cytokines that further influence bone growth via the Wnt/*β*-catenin pathway [25]. In the present study, we have developed a 3D *in vitro* breast cancer bone metastatic model using patient-derived breast cancer cells replacing primary cell lines to predict the metastasis of a patient's breast cancer cells to bone originating from the primary site of the breast. The isolated NT013 and NT023 breast cancer cells from the patient specimens were characterized as hormone-positive and triple-negative, respectively. In line with our previous results [26], we observed the occurrence of MET in both patient-derived breast cell lines in the presence of bone due to the upregulation of E-cadherin and the downregulation of N-cadherin levels. However, we also noticed a dissimilarity in fold change of E-cadherin levels in NT013 and NT023 breast cancer cells grown in the bone microenvironment. The possible reason for a variation in fold change of E-cadherin levels can be attributed to inherent low levels of E-cadherin expression in NT023 that upregulated substantially in the presence of bone microenvironment. In contrast, NT013 cells inherently exhibit a high E-cadherin expression; thus, fold change was not so high. MDAMB231 cells also express low E-cadherin levels inherently [54]. Previously we observed that MDAMB231 cells formed loose aggregates (with poor cell-cell interactions) in the presence of bone because fold change in upregulated E-cadherin levels was not so high [25]. However, in the present study, we observed that NT023 cells formed clustered tumors (with moderate cell-cell interactions) in the presence of bone, suggesting that relatively high E-cadherin levels of NT023 cells stimulate them to form tumors.

The Wnt/*β*-catenin pathway has been well-known for regulating bone formation *in vivo* and osteoblast differentiation *in vitro* [55, 56]. Our results showed that excessive release of ET-1 and DKK-1 by hormone-positive NT013 cells and triple-negative NT023 cells stimulated and abrogated the Wnt/*β*-catenin pathway, respectively. Our previous results also revealed a similar trend by hormone-positive MCF-7 and triple-negative MDAMB231 cells where stimulation and abrogation of the Wnt/*β*-catenin pathway occurred by ET-1 (released by MCF-7) and DKK-1(released by MDAMB231), respectively [25]. Thus, our current results are in good agreement with our previous studies [25] and the reported literature [49, 50]. We have also demonstrated upregulation in OCN levels in the presence of ET-1, indicating excessive bone formation due to increased hMSCs osteogenesis, while downregulation in OCN levels due to the inhibitory effect of DKK-1 leading to inhibited bone formation, in line with our results of primary cell lines [25] and reported studies on bone formation *in vivo* [49, 57–59].

In summary, our 3D *in vitro* model showed both excessive and inhibitory bone growth by the cytokines released from patient-derived cell lines NT013 and NT023 exhibiting different cell characteristics by altering the physiological Wnt/*β*-catenin signaling pathway in a healthy bone. Therefore, this model is suitable for investigating the metastases of cancer to bone and underlying signaling mechanisms during bone metastasis. For more advancement or to better recapitulate bone metastases, we have planned to utilize the patient's hMSCs for model development. In addition, future studies are designed to screen potential drugs to target bone metastasized breast cancer.

## 5. Conclusion

A better understanding of the complex interactions between breast cancer cells and the bone microenvironment is of paramount importance for improving the outcome for late-stage breast cancer patients. One of the critical challenges associated with poor prognosis is the lack of reliable models for studying breast cancer at its advanced stage. In the present study, we utilized a 3D *in vitro* nanoclay-based breast cancer bone metastatic model, previously developed using primary breast cancer cell lines, to investigate the effect of patient-derived breast cancer cells on bone growth. We demonstrate that patient-derived breast cancer cells retain their idiosyncratic characteristics after isolating, using the most efficient method for cancer cell isolation from solid tumors. The model can mimic the MET process of breast cancer metastasis and reveal excessive and inhibitory bone growth by breast cancer cell lines of different characteristics via Wnt/*β*-catenin signaling, mimicking bone lesions observed in breast cancer patients in their late stages. The 3D *in vitro* breast cancer models using patient-derived cells recapitulate the metastatic ability of breast cancer cells to bone. However, future studies are planned to utilize the patient-derived MSCs to develop bone on these scaffolds for more advancement. These models could be a viable tool for future breast cancer studies, including investigating metastatic molecular mechanisms and screening novel drugs.

## Data Availability

The patient data used to support the findings of this study are restricted by the IRB in order to protect patient privacy and are only available to researchers in de-identified form.

## Supplementary Materials

Supplementary Materials
*Table S1.* The details of primary antibodies and their dilutions used for immunofluorescence Staining. *Table S2.* The primer sequence was used for the quantitative reverse transcription-polymerase chain reaction (qRT-PCR) experiment.
