# Supplementary material for: Patient-Derived Breast Cancer Bone Metastasis In Vitro Model Using Bone-Mimetic Nanoclay Scaffolds
Source: J Tissue Eng Regen Med. 2023 Mar 11;2023:5753666. doi: 10.1155/2023/5753666 (PMC11919058; doi:10.1155/2023/5753666)
Supplement: Supplementary Materials — Table S1. The details of primary antibodies and their dilutions used for immunofluorescence Staining. Table S2. The primer sequence was used for the quantitative reverse transcription-polymerase chain reaction (qRT-PCR) experiment. [file 5753666.f1.docx]

**Supplementary data**

**Patient-Derived Breast Cancer Cells for Nanoclay-Based 3D *In Vitro* Bone Metastatic Breast Cancer Model**

Haneesh Jasuja^#^, Farid Solaymani Mohammadi^§,^ Jiha Kim^§^, Anu Gaba^¶^ Dinesh R Katti^#^, Kalpana S Katti^#^*

^#^ Department of Civil Construction and Environmental Engineering

^§^Department of Biological Sciences,

North Dakota State University, Fargo ND 58108, U.S.A.

^¶^ Sanford Roger Maris Cancer Center, Fargo, ND 58102, U.S.A.

* Corresponding Author

[Kalpana.katti@ndsu.edu](mailto:Kalpana.katti@ndsu.edu) , Ph: 701-231-9504

**Table S1.** The details of primary antibodies and their dilutions used for immunofluorescence Staining

| **Primary Antibodies** | **Company** | **Catalog Number** | **Dilutions** |
| --- | --- | --- | --- |
| Progesterone | Cell signaling | 8757S | 1:800 |
| Estrogen | Cell signaling | 13258S | 1:100 |
| HER2 | Cell signaling | 2165S | 1:100 |
| E-Cadherin | Cell signaling | 3195S | 1:200 |
| Vimentin | Abcam | ab8978 | 1:500 |
| CK19 | Abcam | ab52625 | 1:100 |
| EpCAM | Abcam | ab71916 | 1:200 |

**Table S2.** The primer sequence used for the quantitative reverse transcription-polymerase chain reaction (qRT-PCR) experiment

| Gene | Forward primer | Reverse primer |
| --- | --- | --- |
| GAPDH | 5′-CAT CTT CTT TTG CGT CGC CA-3′ | 5′-TTA AAA GCA GCC CTG GTG ACC-3′ |
| E-Cadherin | 5’‐AAG TGA CCG ATG ATG AT‐3’ | 5’‐ CTC TGT CCA TCT CAG CG‐3’ |
| N-Cadherin | 5’‐AGG GTG GAC GTC ATT GTA GC‐3’ | 5’‐CTG TTG GGG TCT GTC AGG AT‐3’ |
| Wnt-5A | 5′-TCT CAG CCC AAG CAA CAA GG-3′ | 5′-GCC AGC ATC ACA TCA CAA CAC-3′ |
| β-catenin | 5′-GGC AGC AAC AGT CTT ACC-3′ | 5′-TCC ACA TCC TCT TCC TCA-3′ |
| VEGF | 5’‐ GAC AAG AAA ATC CCT GTG GGC ‐3’ | 5’‐ AAC GCG AGT CTG TGT TTT TTG C ‐3’ |
| OCN | 5′-GTG ACG AGT TGG CTG ACC-3′ | 5′-TGG AGA GGA GCA GAA CTG G-3′ |
